# Prioritisation and Network Analysis of Crohn's Disease Susceptibility Genes

**DOI:** 10.1371/journal.pone.0108624

**Published:** 2014-09-30

**Authors:** Daniele Muraro, Douglas A. Lauffenburger, Alison Simmons

**Affiliations:** 1 Weatherall Institute of Molecular Medicine, University of Oxford, Oxford, United Kingdom; 2 Department of Biological Engineering, Massachusetts Institute of Technology, Cambridge, Massachusetts, United States of America; University of Chicago, United States of America

## Abstract

Recent Genome-Wide Association Studies (GWAS) have revealed numerous Crohn's disease susceptibility genes and a key challenge now is in understanding how risk polymorphisms in associated genes might contribute to development of this disease. For a gene to contribute to disease phenotype, its risk variant will likely adversely communicate with a variety of other gene products to result in dysregulation of common signaling pathways. A vital challenge is to elucidate pathways of potentially greatest influence on pathological behaviour, in a manner recognizing how multiple relevant genes may yield integrative effect. In this work we apply mathematical analysis of networks involving the list of recently described Crohn's susceptibility genes, to prioritise pathways in relation to their potential development of this disease. Prioritisation was performed by applying a text mining and a diffusion based method (GRAIL, GPEC). Prospective biological significance of the resulting prioritised list of proteins is highlighted by changes in their gene expression levels in Crohn's patients intestinal tissue in comparison with healthy donors.

## Introduction

Biological functions are rarely a consequence of the activity of a single molecule and arise from the interactions between multiple components of biological systems. Since the completion of the human genome project in 2003, high-throughput techniques have generated a large amount of molecular-interaction data in the human cells. The need to analyse the role of associated interaction networks at a system-wide level, rather than focusing on single interactions, led to a change in perspective in the investigation of biological systems and to the development of Systems Biology approaches [1]. During the past decade, significant contributions have been made to curate databases of validated network maps at different levels (protein-interaction, regulatory, metabolic and RNA networks), these often comprising thousands of nodes and links [2], [3]. Investigation of networks of such dimension cannot be easily performed by intuitive reasoning and quantitative approaches are needed to explore their emerging properties more objectively. Recent progresses in network theory have encouraged the application of network based approaches in the study of molecular interaction networks. Although incompleteness in knowledge should suggest caution as these networks are a proxy of the actual interactome, integration with independent functional data may support the biological viability of their topology [4].

When a network-based viewpoint is applied to disease, the disease phenotype is associated with global perturbed networks instead of single failing components [5], [6]. Starting from the underlying assumption that a disease is rarely a consequence of abnormality in single genes, but depends on the indirect perturbation of an interaction network, it should be clarified whether genes and proteins associated with disease are placed randomly in the interactome, or there are correlations between their function and their network topology [7]. Understanding how defects in such networks influence the progression of disease may provide useful information when selecting targets for drug development.

Genetic studies have revealed numerous susceptibility gene variants in common diseases such as Crohn's, but the function of individual gene variants in disease induction remains unclear. Here we use a list of Crohn's susceptibility genes to prioritise genes to serve as a seed to define a putative Crohn's disease network. We then use graph theory to probe hypotheses about its topological structure and to analyse how proteins implicated as being linked to Crohn's disease by this network may relate with their neighbours in the rest of the proteome. Biological relevance of the prioritised list and of its associated interactions is supported by microarray and functional classification data.

This article is organised as follows. First, we prioritise a list of candidate disease genes obtained from literature GWAS reports by applying both a diffusion-based and a text-mining approach. The relevance of our prioritised list is next examined by comparison with differentially expressed genes in biopsies from patients with Crohn's disease. We then build a proteome interaction network of the associated prioritised proteins and we investigate its topological, functional features and relationships with other proteins in the proteome. Correlation between topological localisation and functional role supports the biological relevance of the datasets interactions. The network associated with disease shows enrichment in hubs nearest neighbours and topological segregation of the prioritised list. In the light of our observations, we conclude by highlighting proteins in the network associated with disease with noteworthy topological and functional properties that may warrant further experimental investigation.

## Results and Discussion

In what follows we prioritise a list of candidate genes associated with Crohn's disease and test its enrichment among the set of differentially expressed genes in patients affected by Crohn's disease. We then build a molecular interaction network from this list and test correlations between the network topology and its functional organisation. In each section we first provide a brief review of the relevant methods, we then describe in more detail our particular application. The technical details of the methods applied are described either in the section Methods or in (Information S1).

### Prioritisation of genes associated with Crohn's disease

Genome-Wide Association Studies have identified a large number of candidate disease genes for Crohn's but the role of each in disease pathogenesis is unclear [8]. In order to reduce the number of candidate genes and to identify the disease module, several tools from bioinformatics and biomathematics have been proposed. Such methods rely upon different assumptions and can be classified in three main categories as pairwise, neighbourhood and diffusion based methods [9]. Pairwise methods assume that proteins associated with disease tend to directly interact with each other. In this category, linkage methods select genes located in the linkage interval of genes whose protein product is a first neighbour of proteins associated with disease. Other pairwise methods analyse relatedness between two genes by applying text mining and assessing a score to the association depending on the degree of similarity in the text describing them within article abstracts [10]. Neighbourhood based methods rely upon the hypothesis that cellular components associated with the same disease tend to cluster together [8].

In diffusion based methods, random walkers are released from a set of known disease genes and diffuse along the links of the proteome; in such a way, nodes that are more connected to disease proteins are more frequently visited and prioritised [11]. All of these methods depend on the topological structure of the interactome; but, while linkage and neighbourhood based methods rely upon a particular topological metric, such as pairwise or nearest interactions, diffusion based methods adopt the full information of the network topology. Diffusion-based methods have been recently applied and shown to achieve the state-of-the-art predictive performance [12], [13], [14], [15]; in addition, combining predictions made by different methods in a 'consensus method' yielded to Pareto optimal performance in the precision-recall objectives [15].

**Figure 1:**
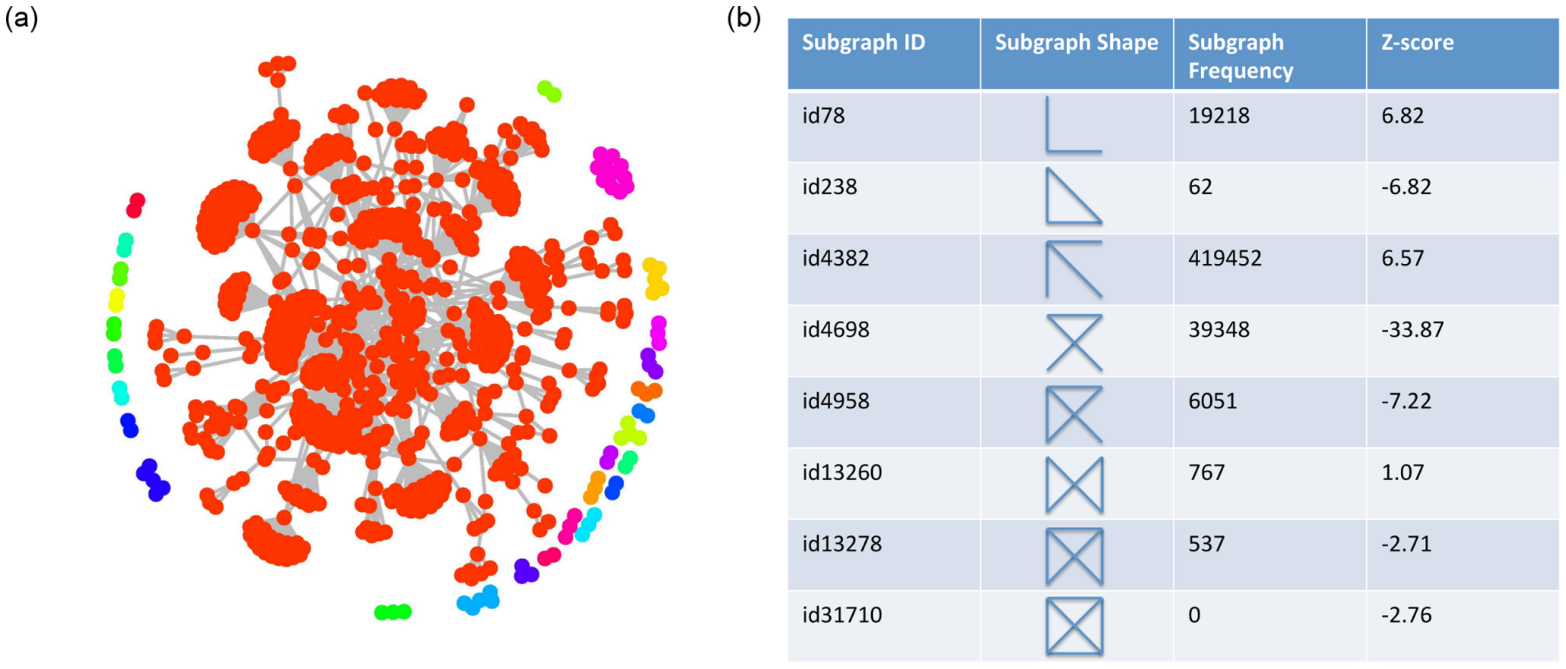
Network associated with Crohn's disease and motifs. (a) Representation of the protein interaction network obtained by prioritisation. The network presents 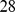 connected components, each one being highlighted using a different colour. The giant component, namely the connected subgraph that contains the majority of the entire graph's nodes, is shown in red. (b) Sub-graphs frequency and z-scores in the network associated with Crohn's. Considering the threshold 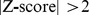, subgraphs id78 and id4382 are over-represented (motifs), whereas subgraphs id238, id4698, id4958, id13278, id31710 are under-represented (anti-motifs).

Accounting for the results of this comparative analysis, we selected 171 SNPs and 354 genes associated with Crohn's disease from the Catalog of Published Genome-Wide Association Studies [16] and in a recent published GWAS by Jostins et al. [17] and we performed prioritisation of these genes using both a diffusion based method and a pairwise text mining algorithm (see Methods section). We finally selected a consensus list from the results of the prioritisation algorithms, together with the training set of known genes, to obtain a sub-list of 99 genes. From this list we built a sub-network associated with Crohn's disease by selecting all interactions containing at least one protein identified by prioritisation; in such a way, we also considered indirect interactions among proteins associated with disease, as suggested by Rossin et al. [8]. This sub-network is shown in Figure 1. The list of the prioritised proteins and the interactions in the network associated with disease are reported in an Excel workbook in the (Workbook S1). Support for involvement of this protein network as being implicated in Crohn's disease related inflammation was then obtained by comparing our list with genes whose expression has been identified as being differentially regulated in intestinal tissue from patients with Crohn's. We used publicly available microarray data from a study whose aim was to investigate differential intestinal gene expression in patients with Crohn's disease (CD) and controls (see Methods section). As a result of this selection we found that 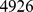 genes of the 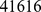 measured in the microarray were differentially expressed of which 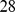 were part of the 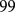 prioritised genes. A Fisher's exact test shows enrichment in differentially expressed genes among the prioritised ones with p-value equal to 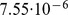, thus supporting the association of the prioritised list to Crohn's disease. Interestingly not all the genes of the training set, although associated with Crohn's disease, are differentially expressed; this suggesting that differential expression should be combined with other criteria, such as functional and topological, to support selection of candidate proteins as associated with disease. The list of Entrez IDs of the prioritised list together with their p-values is reported in Workbook S1.

### Topological characterisation of the network associated with Crohn's disease

We analysed the global and local topological organisation of the sub-network that we have built in the previous section. Characteristic graph-theoretical distributions and metrics show signatures of hierarchical modularity and preferential attachment; these properties resemble the ones of other biological networks, this supporting the biological viability of the network that we associated with Crohn's disease (see Information S1, Figure S1 and Table S1). The density of this network is approximately three times higher than in the NCBI proteome network suggesting a higher tendency of the disease proteins to interact among themselves than among proteins that are not associated with disease.

Since disease is often caused by perturbation in the communication between bio-molecules [18], [19], investigating how such changes at the local level can affect the network structure may provide insight into its robustness and highlight which components are critical to maintain a correct functioning. Analysis of network robustness by node removal (failure-attack tolerance) shows robustness to removal of nodes with low degree and susceptibility to deletion of highly connected nodes; this reflects the key role played by hub proteins in maintaining the connectivity of this biological network. A detailed description of this analysis is reported in the Information S1 (see also Figures S2 and S3).

We then investigated if proteome hubs are over-represented in the network associated with Crohn's and we analysed if the number of hubs in the list of prioritised proteins is over-represented when compared to the total number of hubs in the NCBI proteome. The p-value obtained by a hypergeometric distribution does not show a significant over-representation (see Table 1). We then considered the list of proteins in the network associated with Crohn's, including the first neighbours of the prioritised list, and analysed their over-representation in a similar manner; in this case, hub over-representation is significant, suggesting Crohn's disease susceptibility genes tend to directly interact with proteome hubs.

**Table 1:** Hubs distribution.

Proteins list	N. proteins	N. Hubs	p-value
NCBI Human PPI network proteins	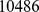	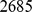	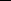
Prioritised proteins	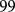	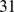	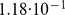
Disease network proteins	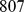	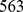	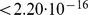

Table summarising the number of hub proteins in the NCBI proteome, in the list of prioritised proteins and in the same list together with their first neighbours (Disease network proteins). Over-representation of hubs is statistically significant when considering first neighbours of the prioritised list (Hypergeometric distribution p-values).

The global features of preferential attachment and hierarchical modularity suggest the presence of sub-graphs characterising the network at a local level. We now address the problem of identifying such topological modules and analysing their potential correlation with proteins associated with disease. More specifically, we searched for over-represented subgraphs (motifs) when compared to randomised versions of the same network. Algorithms for the search of network motifs explore the full combinatorial set of graphs of a given dimension. Since the computational time grows exponentially with graph dimension, small motifs comprising three or four nodes are usually analysed [20]. Several tools have been developed to identify network motifs, such as Mfinder [21], MAVisto [22], FANMOD [23]. A well established tool developed for network motif search is Mfinder [21]. Beginning with a selected edge, Mfinder searches for all the subgraphs of a given dimension comprising it. All the sets of visited nodes are then stored in a hash table, this reducing the searching time as the searching tree is stopped when a set of nodes has been already visited. Motif over-representation is then evaluated by comparing the frequency of motifs in the real network with a set of randomly generated networks. In the default mode random networks preserve the degree distribution of the nodes and are generated using a switching method, namely edges are switched while keeping the number of incoming edges, outgoing edges and mutual edges of each node of the input network. We investigated the presence of motifs and anti-motifs in the network associated with disease by applying Mfinder with the default conditions. Because of the computational time required, we analysed motifs of three or four nodes only and evaluated their over-representation over 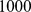 random networks. According to the default Z-score threshold (Z-score = 2), the network associated with Crohn's contains 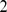 motifs (with motif ids id78 and id4382) and 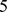 anti-motifs (with motif ids id238, id4698, id4958, id13278, id31710), (Figure 1b). Interestingly, cliques composed of four nodes are under-represented, suggesting that such a high level of connectivity is not likely in realistic biological networks. We then analysed which prioritised proteins were more frequently associated with motifs and we found, in order of frequency, PRDM1, ATF4 and FASLG. Notwithstanding the degree distribution of the network associated with Crohn's was preserved when generating random networks, two of these proteins are highly connected, FASLG being the fourth most connected protein in the prioritised list and ATF4 the thirteenth. ATF4 is also one of the known proteins associated with Crohn's disease, see Table S2.

### Functional classification and topological segregation of enriched categories

Based on the assumption that proteins with similar functional properties interact with one another, protein interaction maps have been frequently used to generate hypotheses on the functional role of proteins of unknown functional classification [24], [25]. A systematic graph-theoretical study built from this premise was proposed in [4] on four datasets that approximate the protein interaction network of yeast *Saccharomyces cerevisiae*. In order to determine how well such datasets characterise the protein interaction network of *Saccharomyces cerevisiae*, the authors investigated the relationship between the topology of the protein interaction maps and the known functional properties of the protein. In all four datasets strong correlations were found between the network's structure and the functional role and sub-cellular localisation of its protein constituents. By measuring the tendency of proteins to interact with other proteins of the same functional or localisation class they concluded that most functional classes appear as relatively segregated sub-networks of the full protein interaction network.

**Figure 2:**
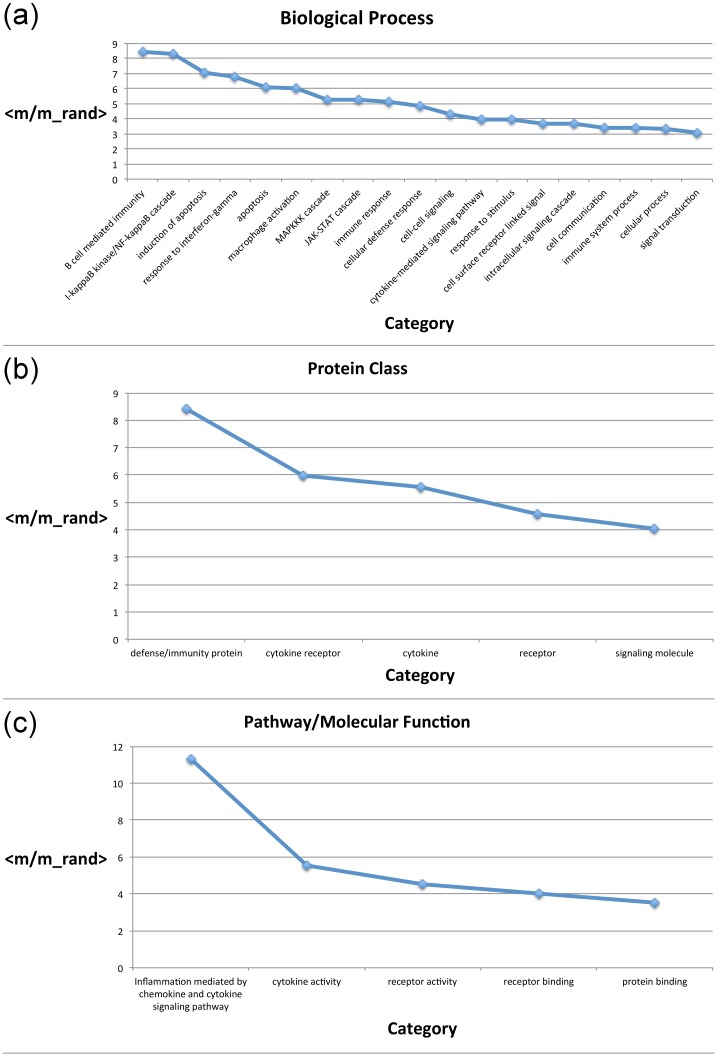
Topological segregation. Series of plots representing the segregation functions of over-represented categories in the network associated with Crohn's sorted from the most to the least segregated category. (a) categories within biological processes; (b) categories within protein classes; (c) categories within molecular functions and pathways.

In the spirit of this analysis, we examined whether the protein network that we associated with Crohn's disease leads to a similar correlation with the functional properties of the prioritised proteins. We performed a functional classification by applying the PANTHER (Protein ANalysis THrough Evolutionary Relationships) Classification System [26]. Here proteins have been functionally classified according to molecular function (the function of the protein by itself or with directly interacting proteins at a biochemical level, e.g. a protein kinase); biological process (the function of the protein in the context of a larger network of proteins that interact to accomplish a process at the level of the cell or organism, e.g. mitosis) or pathway (similar to biological process, but a pathway also explicitly specifies the relationships between the interacting molecules). We asked whether enriched categories presented a correlation with network topology being topologically segregated. Categories comprising less than 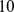 proteins were not considered in this analysis as they are too few to perform a statistical characterisation. Topological segregation was evaluated by calculating the segregation function 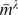 per functional class 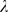 in the enriched categories (see Methods section). This function represents how many times it is more likely that proteins in a particular functional category interact with neighbours belonging to the same category than with proteins randomly placed in the network. The evaluation of the topological segregation is reported in Figure 2. Particularly interconnected classes are the ones related to inflammation ('Inflammation mediated by chemokine and cytokine signalling pathway') and to the immune system ('defense/immunity protein'). Correlation between topology and functional organisation further supports the biological relevance of the network topology. Evaluation of the topological segregation of the prioritised list by Eq. (1) in the Methods section returned a value of 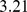 showing tendency of these proteins to aggregate.

## Conclusions

In this work we have prioritised a list of genes associated with Crohn's disease and developed a graph-theoretical analysis of the molecular interaction network resulting from this list. Prioritisation was performed by applying both a diffusion based method (GPEC) [11] and a pairwise text mining algorithm (GRAIL: Gene Relationships Across Implicated Loci) [10] with available software. The relevance of the prioritised list was supported by enrichment in differentially expressed genes in microarray data between biopsies taken from patients with Crohn's disease and healthy controls. By analysing the network associated with Crohn's from a graph-theoretical perspective, we have shown that it presents hierarchical modularity and density higher than in the NCBI proteome network, this suggesting a higher tendency of the disease proteins to interact among themselves than among proteins that are not associated with disease. Finally we have analysed the relationships among the topology of this network and the functional properties of its proteins. To test if prioritised proteins associated with the same functional class are more likely to interact among each other than with other proteins we have calculated their segregation function and we have highlighted a correlation between functional role and their topological location, this being also in agreement with the global modular organisation of the disease network. A small number of the prioritised proteins demonstrated both noteworthy functional and topological properties which are discussed below. STAT3 and JAK2 are present in 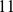 and 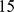 over-represented and topologically segregated functional categories respectively; they interact in the same signaling path 'JAK-STAT cascade', they were both differentially expressed in Crohn's tissue and they are highly interconnected with hubs as first neighbours, besides being highly interconnected proteins themselves in the network associated with disease, see Table 2. Vitamin D receptor (VDR) represents a strong positional candidate susceptibility gene for inflammatory bowel disease (IBD) [27] and is part of the training set (see Table S2); it is highly interconnected in the network associated with disease and also highly interconnected with hubs as a first neighbour (see Table 2); in addition, it is present in 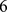 over-represented and topologically segregated functional categories. PRDM1 is the protein which is most frequently present in network motifs and the adjusted p-value associated to its differential expression, although not being under the arbitrary statistical threshold of 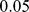, is still significant being 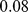; it is also highly interconnected with hubs as a first neighbour (see Table 2). FASLG is present in 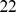 over-represented and topologically segregated functional categories, it is one of the proteins that occur most frequently in network motifs, it is also highly interconnected in the network associated with Crohn's and highly interconnected with hubs as a first neighbour (see Table 2). ATF4 is a protein of the training set and is part of the unfolded protein response (UPR) pathway which has been recently emerged in IBD pathophysiology [28], [29], [30]; it is one of the proteins most frequently associated with network motifs and it is highly interconnected with hubs as a first neighbour (see Table 2). A table listing the over-represented functional categories of the proteins just mentioned is reported in Table S3. Selected proteins combining functional and topological information may constitute candidates to investigate novel interactions between proteins directly associated to a causal mutation and proteins whose perturbation may be indirectly relevant in affecting the disease phenotype.

**Table 2:** Selected proteins.

Protein name	N. neighbours	N. hub neighbours	p-value
STAT3	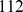	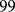	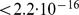
JAK2	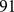	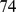	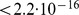
VDR	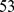	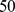	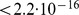
PRDM1	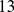	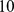	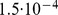
FASLG	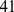	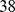	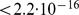
ATF4	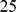	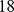	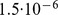

Table summarising the number of hub first neighbours in the selected proteins listed in section 'Results and discussion'. P-values represent the probability that the number of neighbour hubs is due to random choice and are calculated using a Fisher's exact test which compares the total number of hubs in the NCBI proteome with the number of hubs in the neighbours of the selected proteins. Of the 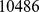 proteins listed in the NCBI protein interaction network 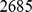 have a number of first neighbours which is strictly higher than the average; connectivity with these hubs is over-represented for the 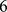 proteins presented.

## Methods

### Prioritisation algorithms

171 SNPs and 354 genes associated with Crohn's disease were downloaded from the Catalog of Published Genome-Wide Association Studies [16] and from a recent published GWAS by Jostins et al. [17]. Genes and SNPs association is given by the locus list defined by the NHGRI GWAS catalogue [16], whose annotation was applied by Jostins et al., and that reports the strongest SNP and genes reported by the author(s) of the publication per locus window. Prioritisation was derived by the consensus of two algorithms, namely a diffusion based method (GPEC) [11] and a pairwise text mining algorithm (GRAIL: Gene Relationships Across Implicated Loci) [10] using as input SNPs rs numbers and Entrez IDs respectively with available software. GRAIL has two input sets of disease regions in the form of genomic regions around associated SNPs: a collection of seed regions and a collection of query regions. Genes in query regions are evaluated for relationships to genes in seed regions, and query regions are then assigned a significance score. When examining a set of regions for relationships between implicated genes, as in this case, the query regions and the seed regions are identical. GRAIL ranks genes by text similarity calculating gene relatedness as the degree of similarity in the text describing them within PubMed article abstracts; the algorithm then assigns a p-value to each gene by evaluating the number of other disease regions with related genes. By querying all human genes within the database, GRAIL associated 156 of the 171 SNPs to 174 genes with a p-value less than 0.1. We then applied GPEC on the list of genes reported from the collection of GWAS as follows. Prioritisation with GPEC was performed through a random walk with restart algorithm along a gene or protein relationship network. Nodes in the network were represented by Entrez Gene IDs, UniProt ACs, or official symbols for genes and proteins. A set of training genes, whose role in disease is verified in the literature, was specified together with a set of candidate genes which was defined as the list of genes associated with Crohn's disease from GWAS. The list of the candidate genes is reported in Workbook S1, whereas the list of training genes, together with a list of literature references, is listed in Table S2. A human protein-protein interaction network was downloaded from the NCBI Entrez Gene FTP site (ftp://ftp.ncbi.nlm.nih.gov/gene/GeneRIF/interactions.gz) which integrates three databases: Biomolecular Interaction Network Database [31], Biological General Repository for Interaction Datasets [32], Human Protein Reference database [33]. As a result a network of 10,486 genes and 50,791 interactions was built and employed to define the graph on which the random walk was defined. Random walkers were then initialised in the set of training genes and allowed to diffuse along the protein interaction network until they reached a steady state, which is numerically approximated by repeating the iterations until the difference between the vector of probabilities at time 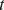 and at time 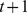, where the *i*-th element represents the probability of the walker being at node 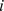 at a fixed time, is smaller than a threshold value (whose default value is set to 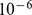). As a result of the GPEC algorithm run a set of 212 genes were identified at steady state. We finally selected a consensus list from the results of the prioritisation algorithms, together with the training set of known genes, to obtain a sub-list of 99 genes.

### Microarray dataset

The microarray dataset analysed is available at Gene Expression Omnibus (http://www.ncbi.nlm.nih.gov/geo/ accession number GSE20881). 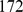 biopsies from CD and control subjects were studied. Endoscopic biopsies were taken at ileocolonoscopy from four specific anatomical locations, these being terminal ileum, sigmoid colon, ascending colon, descending colon [34]. The groups of CD and healthy samples were compared in order to identify genes that are differentially expressed across experimental conditions using the interactive web tool GEO2R (http://www.ncbi.nlm.nih.gov/geo/geo2r). GEO2R performs comparisons on original submitter-supplied processed data tables using the GEOquery and limma R packages from the Bioconductor project (http://www.bioconductor.org). The Benjamini and Hochberg false discovery rate method was selected by default to adjust p-values for multiple testing. We used these values as the primary statistics by which to interpret results, selecting as differentially expressed genes those whose p-value was less than 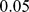.

### Categories enrichment

Enrichment was performed by applying a statistical over-representation test to the prioritised proteins using as a reference list the set of all genes in the genome classified in the PANTHER database. Each list is compared to the reference list using the binomial test [35] for each molecular function, biological process, or pathway term in PANTHER; Bonferroni correction is applied for multiple testing. PANTHER mapped 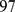 of the 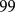 disease proteins into different categories and assigned a p-value to each category. Categories with a p-value minor than 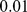 were considered over-represented; their chart representations are reported in Figures S4–S7 and their lists in Workbook S1. Classification by cellular component returned a number of classified proteins that was too low for a statistical analysis, for completeness these are reported in Workbook S1.

### Evaluation of the topological segregation

The presence of topological segregation was evaluated by calculating its segregation function for each enriched category; this is defined as follows. Given a protein 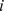 belonging to the functional class 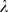 the segregation function is given by 
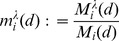
where 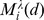 denotes the number of proteins at distance 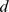 from protein 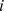 and belonging to the functional class 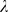 and 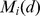 denotes the total number of proteins at distance 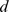 from protein 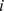. We then denote by 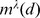 the average of all 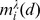 belonging to the same class 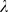: 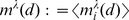
If proteins of a functional class 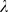 were randomly distributed, then (see [4]) 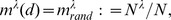
for any 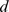, where 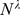 denotes the total number of proteins belonging to the functional class 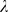 and 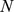 is the total number of proteins in the protein network. Defining 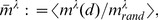
(1)where the average is taken over the distance, a random distribution would return 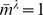.

## Supporting Information

Figure S1 **Topological distributions.** Characteristic graph-theoretical distributions of the NCBI human protein-protein interaction network and of the protein interaction network obtained by prioritisation. (a), (b) average clustering coefficient distributions; (c), (d) topological coefficient distributions. A formal definition of these distributions is reported in the Appendix.
(TIFF)

Figure S2 **Failure-attack tolerance to node removal.** Series of plots representing how the number of interactions and the number of secondary extinctions vary when removing nodes randomly (black circles), from the highest to the lowest degree (red circles) and from the lowest to the highest degree (green circles). (a) Number of interactions in the network associated with Crohn's against percentage of removed nodes; (b) Number of secondary extinctions in the network associated with Crohn's against percentage of removed nodes; (c) Number of interactions in a random network against percentage of removed nodes; (d) Number of secondary extinctions in a random network against percentage of removed nodes.
(TIFF)

Figure S3 **Failure-attack tolerance to SNP removal.** Plots representing how the number of interactions varies when removing nodes associated with the SNPs locus windows (blue) and when removing the same number of nodes from the highest to the lowest degree (red circles) and from the lowest to the highest degree (green circles).
(TIFF)

Figure S4
**Enriched biological processes.** Chart summarising the biological precesses that are enriched in the prioritised list of proteins. P-value threshold was set to 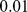.(TIFF)

Figure S5
**Enriched protein classes.** Chart summarising the protein classes that are enriched in the prioritised list of proteins. P-value threshold was set to 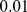.(TIFF)

Figure S6
**Enriched molecular functions.** Chart summarising the molecular functions that are enriched in the prioritised list of proteins. P-value threshold was set to 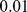.(TIFF)

Figure S7
**Enriched pathways.** Chart summarising the pathways that are enriched in the prioritised list of proteins. P-value threshold was set to 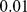.(TIFF)

Table S1
**Topological metrics.** Table summarising the topological properties of the disease network and of 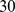 Erdős-Rényi networks with the same number of nodes and edges. All the listed properties in the disease network are significantly different from random with p-values, calculated from z-scores, smaller than 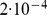. 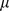 and 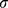 are respectively mean values and standard deviations of the graph metrics.(PDF)

Table S2 **Training set.** Table listing the Entrez IDs included in the training set with their literature references.
(PDF)

Table S3 **Segregated enriched categories.** Table summarising the segregated enriched categories containing STAT3, JAK2, VDR, FASLG (see section 'Results and discussion' in the main text). ATF4 and PRDM1 are not reported not being present in such categories.
(PDF)

Workbook S1
**Network associated with Crohn's disease and enrichment tables.** Workbook containing the candidate SNPs and Entrez IDs (Sheet 1), the prioritised Entrez IDs (Sheet 2), the network associated with Crohn's disease (Sheet 3), the NCBI proteome network (Sheet 4), the interactions among the proteins associated with the 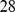 prioritised and differentially expressed genes (Sheet 5) and the enrichment tables in biological processes (Sheet 6), protein classes (Sheet 7), molecular functions (Sheet 8), pathways (Sheet 9), cellular components (Sheet 10).(XLS)

Information S1 **Supplementary Text and Supplementary Tables.**
(PDF)
