# Supplementary figures and images for: Prioritisation and Network Analysis of Crohn's Disease Susceptibility Genes
Source: PLoS One. 2014 Sep 30;9(9):e108624. doi: 10.1371/journal.pone.0108624 (PMC4182533; doi:10.1371/journal.pone.0108624)

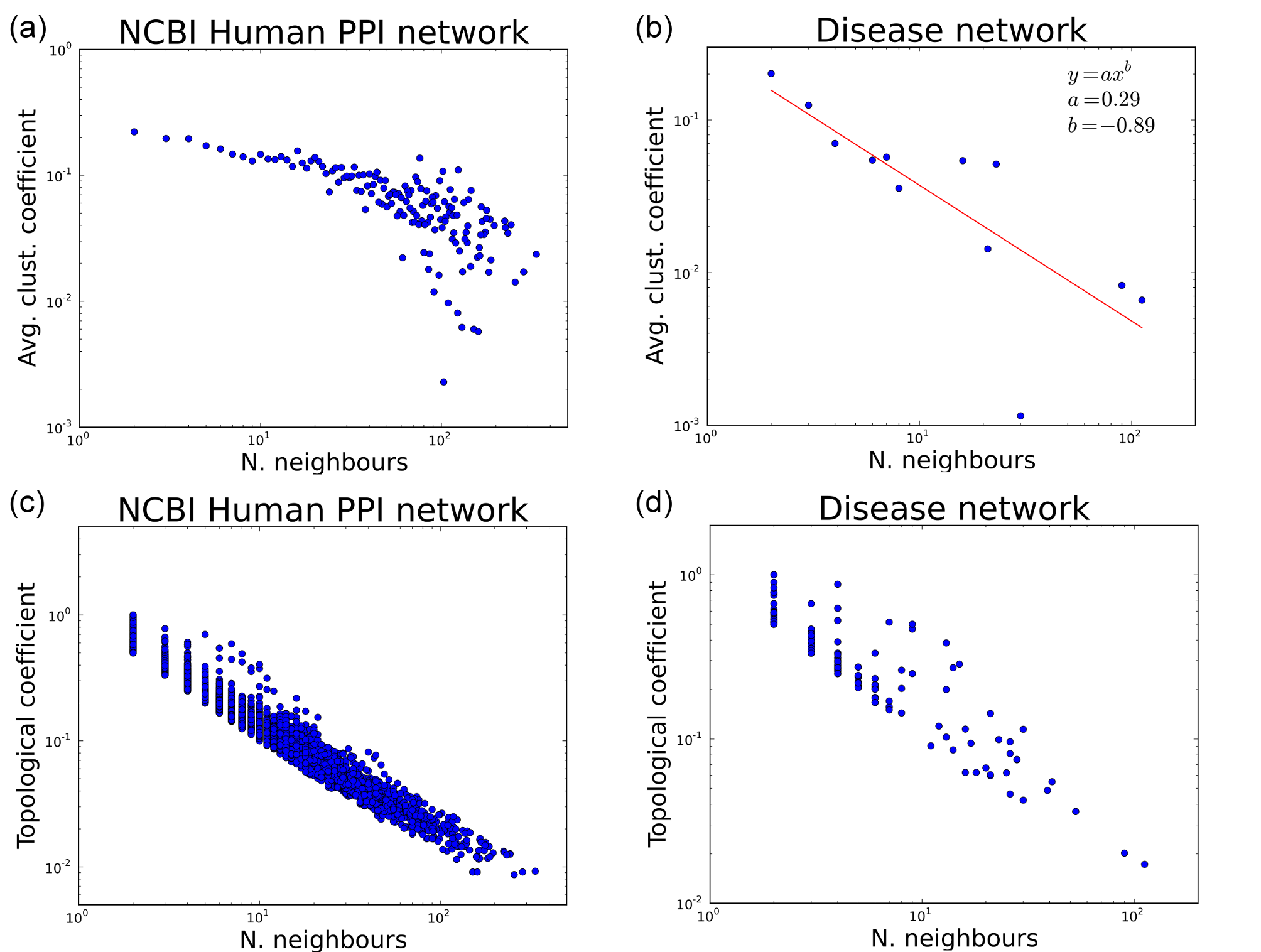

Supplement: Figure S1 — Topological distributions. Characteristic graph-theoretical distributions of the NCBI human protein-protein interaction network and of the protein interaction network obtained by prioritisation. (a), (b) average clustering coefficient distributions; (c), (d) topological coefficient distributions. A formal definition of these distributions is reported in the Appendix. (TIFF) [file pone.0108624.s001.tiff]

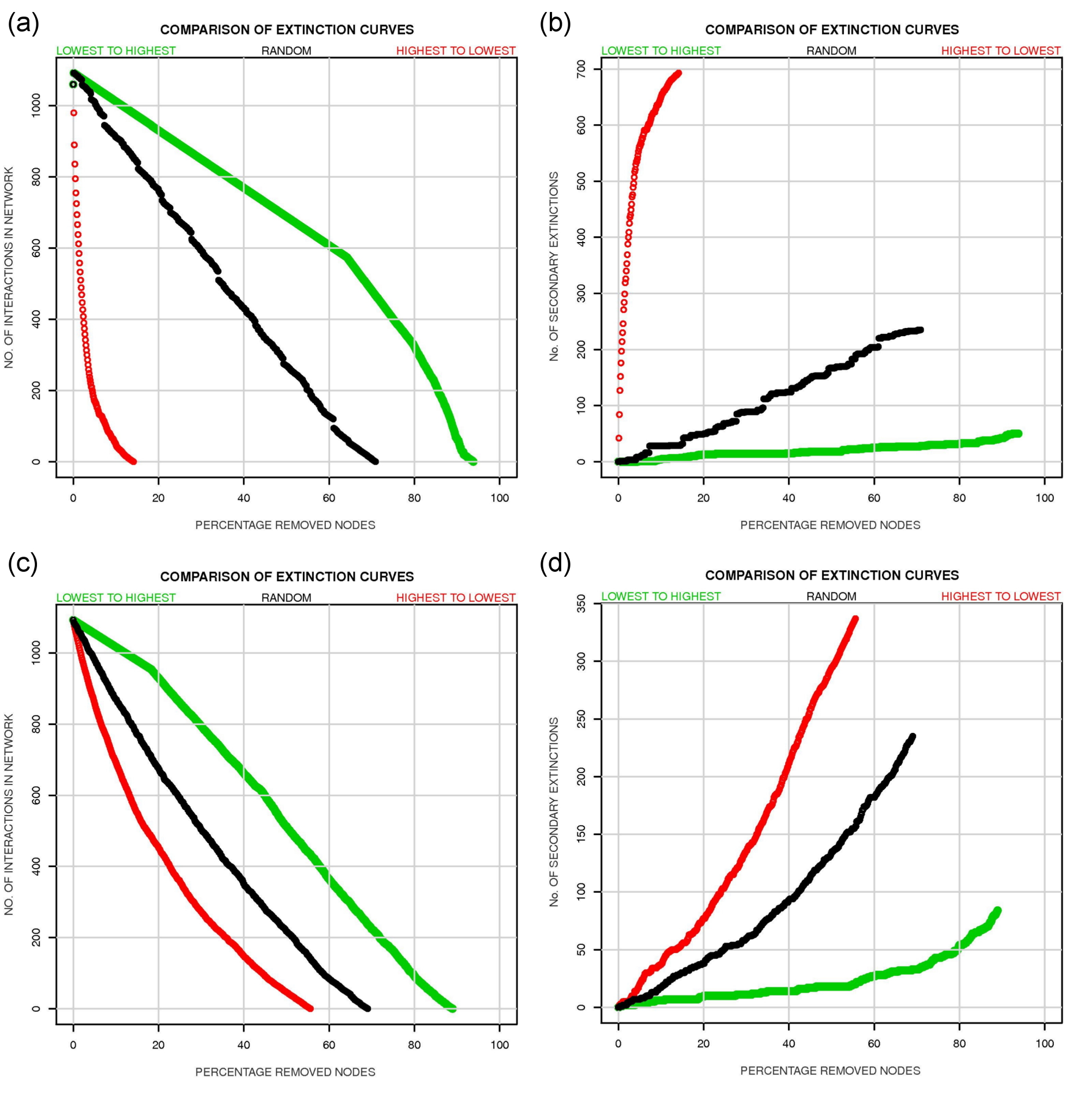

Supplement: Figure S2 — Failure-attack tolerance to node removal. Series of plots representing how the number of interactions and the number of secondary extinctions vary when removing nodes randomly (black circles), from the highest to the lowest degree (red circles) and from the lowest to the highest degree (green circles). (a) Number of interactions in the network associated with Crohn's against percentage of removed nodes; (b) Number of secondary extinctions in the network associated with Crohn's against percentage of removed nodes; (c) Number of interactions in a random network against percentage of removed nodes; (d) Number of secondary extinctions in a random network against percentage of removed nodes. (TIFF) [file pone.0108624.s002.tiff]

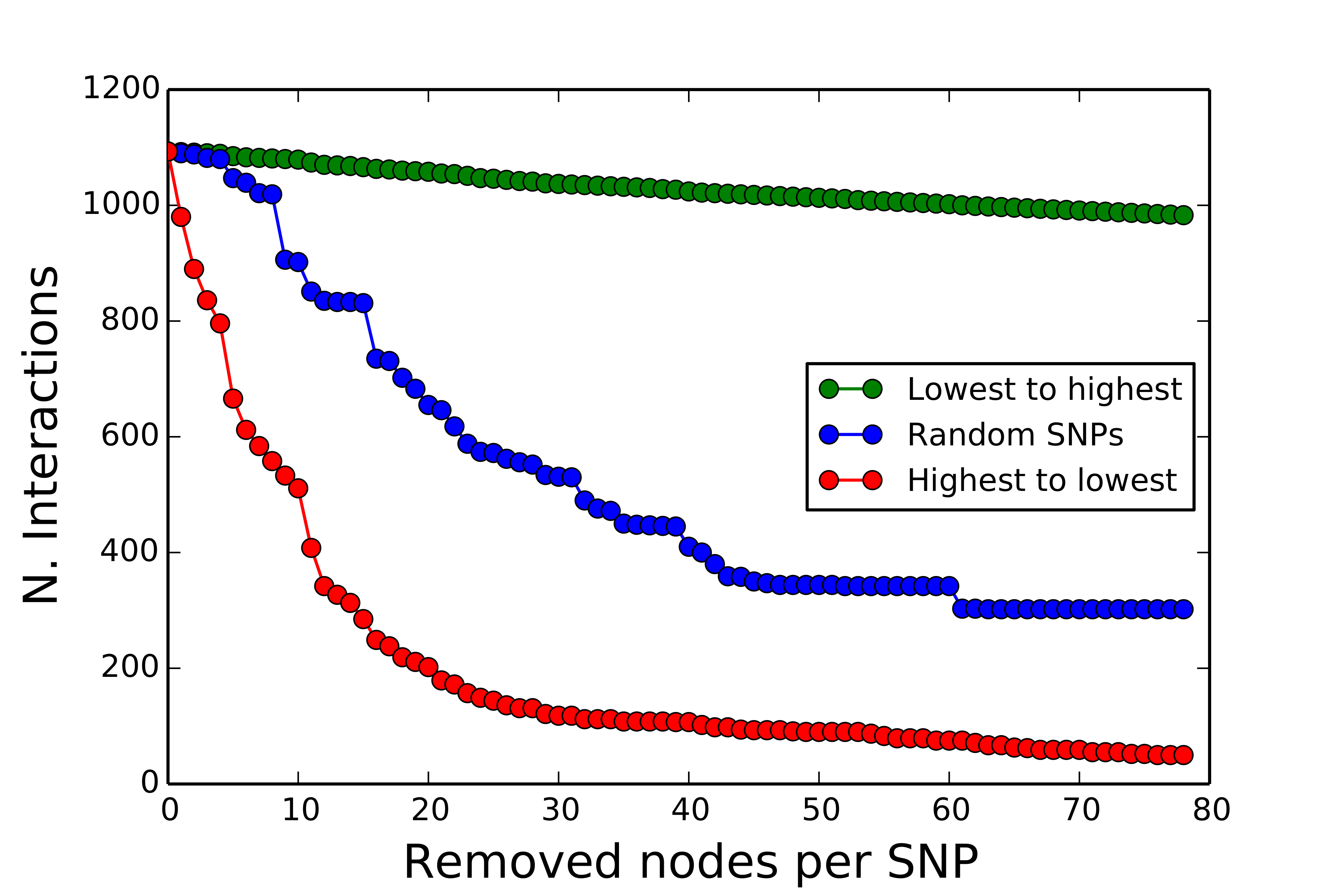

Supplement: Figure S3 — Failure-attack tolerance to SNP removal. Plots representing how the number of interactions varies when removing nodes associated with the SNPs locus windows (blue) and when removing the same number of nodes from the highest to the lowest degree (red circles) and from the lowest to the highest degree (green circles). (TIFF) [file pone.0108624.s003.tiff]

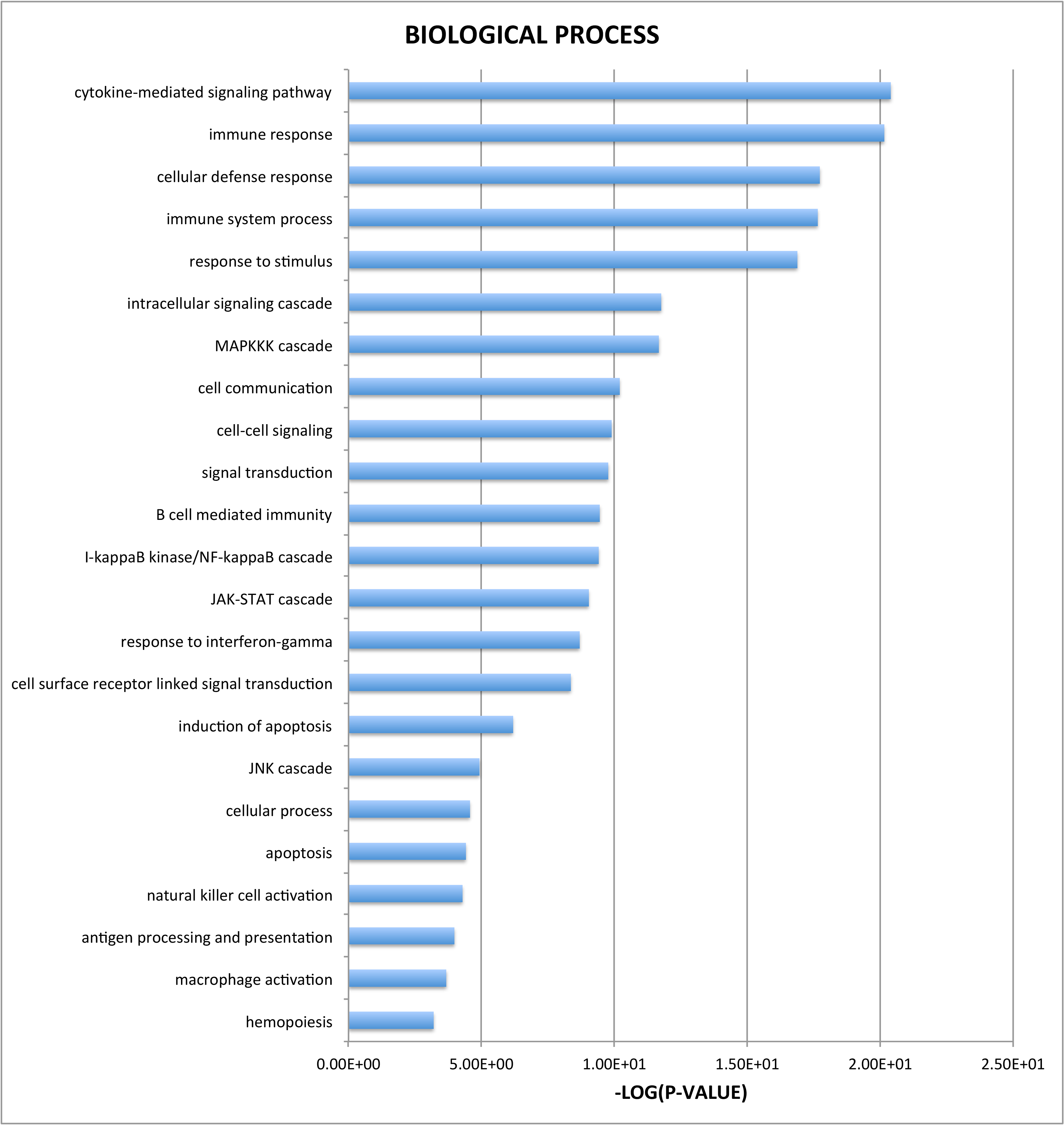

Supplement: Figure S4 — Enriched biological processes. Chart summarising the biological precesses that are enriched in the prioritised list of proteins. P-value threshold was set to . (TIFF) [file pone.0108624.s004.tiff]

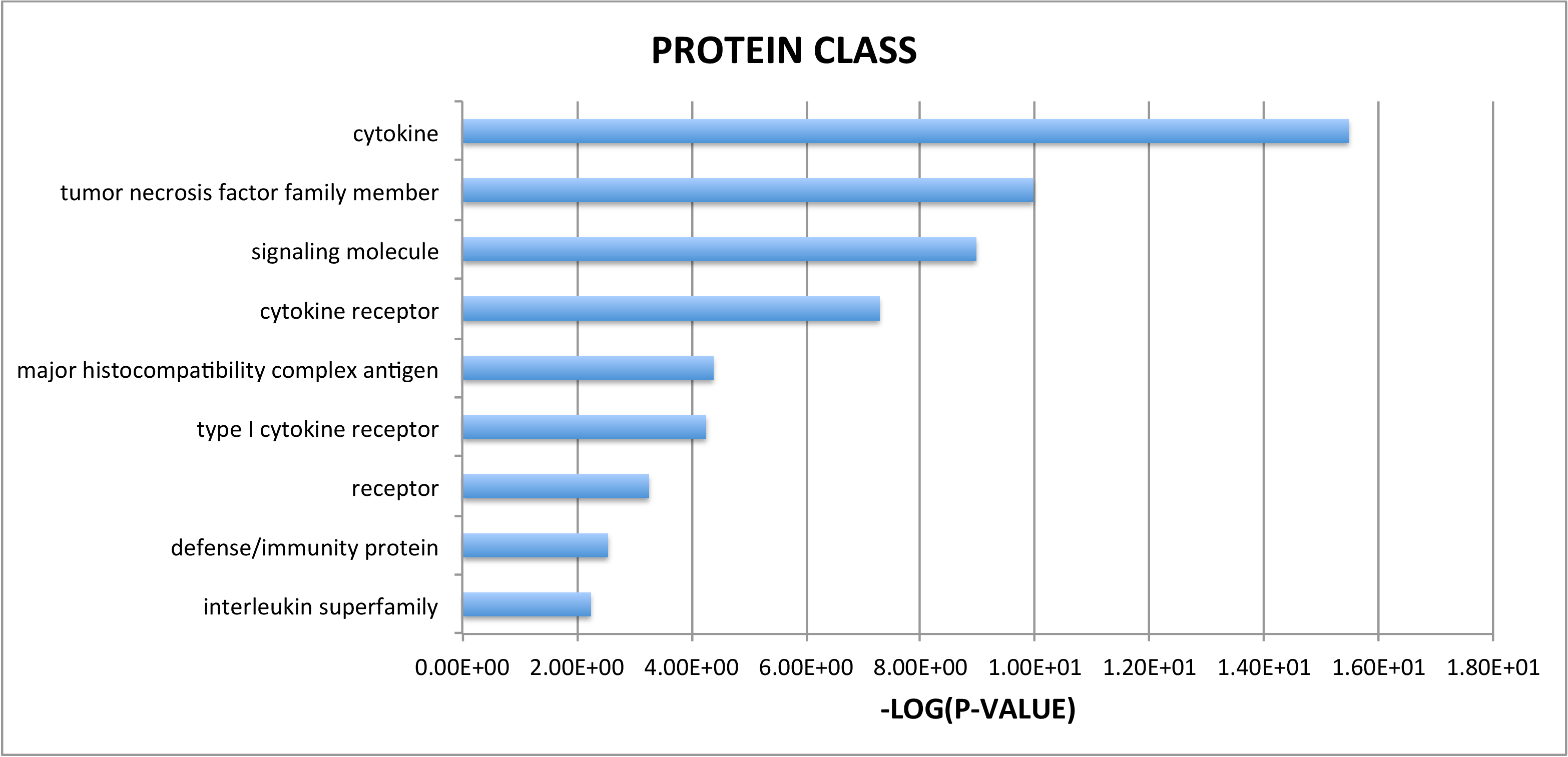

Supplement: Figure S5 — Enriched protein classes. Chart summarising the protein classes that are enriched in the prioritised list of proteins. P-value threshold was set to . (TIFF) [file pone.0108624.s005.tiff]

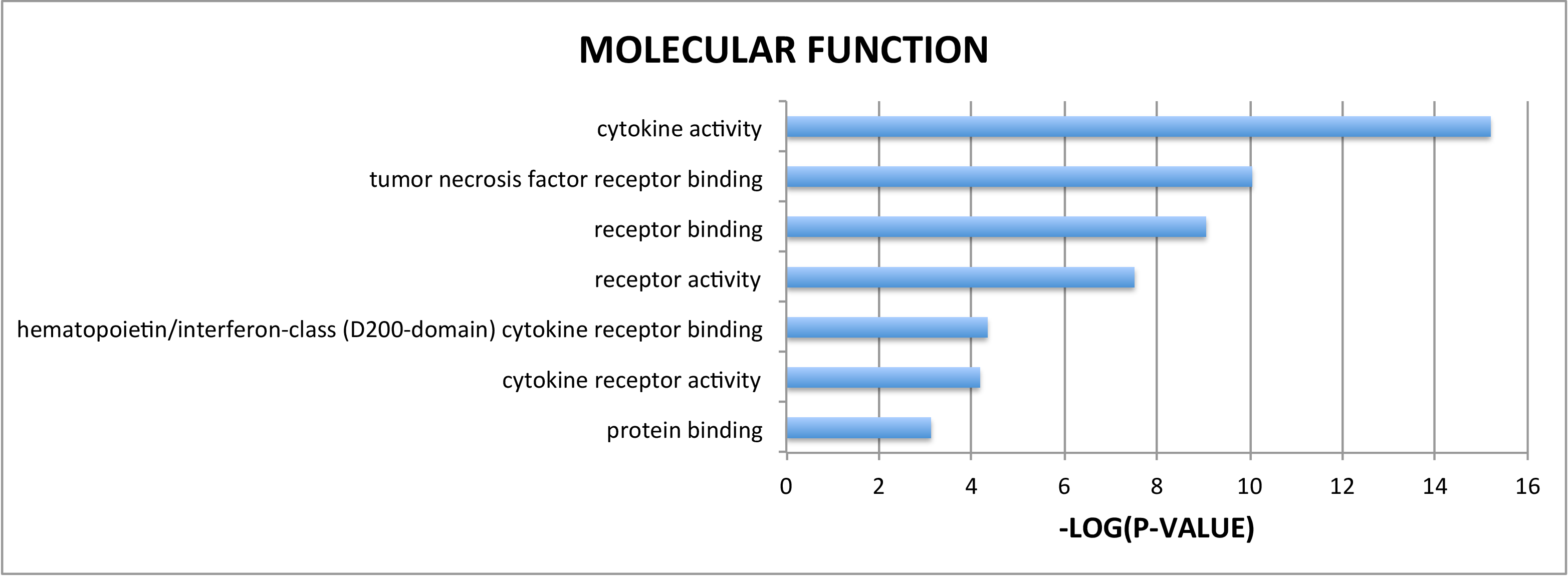

Supplement: Figure S6 — Enriched molecular functions. Chart summarising the molecular functions that are enriched in the prioritised list of proteins. P-value threshold was set to . (TIFF) [file pone.0108624.s006.tiff]

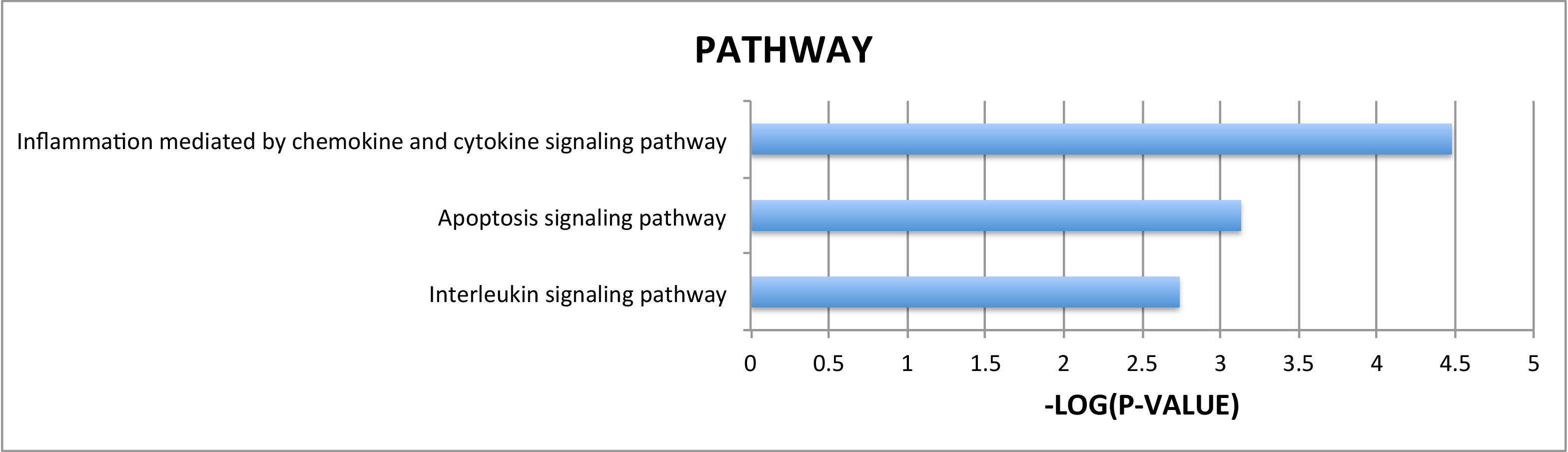

Supplement: Figure S7 — Enriched pathways. Chart summarising the pathways that are enriched in the prioritised list of proteins. P-value threshold was set to . (TIFF) [file pone.0108624.s007.tiff]
