# Supplementary material for: Prioritisation and Network Analysis of Crohn's Disease Susceptibility Genes
Source: PLoS One. 2014 Sep 30;9(9):e108624. doi: 10.1371/journal.pone.0108624 (PMC4182533; doi:10.1371/journal.pone.0108624)
Supplement: Table S1 — Topological metrics. Table summarising the topological properties of the disease network and of Erdős-Rényi networks with the same number of nodes and edges. All the listed properties in the disease network are significantly different from random with p-values, calculated from z-scores, smaller than . and are respectively mean values and standard deviations of the graph metrics. (PDF) [file pone.0108624.s008.pdf]

Table S1

Graph metric	NCBI Human PPI network	Disease network	Random networks ($\mu \pm \sigma$)
Number of nodes	10486	807	807
Number of edges	50791	1093	1093
Network density	$8.817 \cdot 10^{-4}$	$3.361 \cdot 10^{-3}$	$3.361 \cdot 10^{-3}$
Clustering coefficient	$1.239 \cdot 10^{-1}$	$4.446 \cdot 10^{-2}$	$2.499 \cdot 10^{-3} \pm 1.811 \cdot 10^{-3}$
Network diameter	14	10	15 ± 1.365
Average path length	4.071	4.706	$6.517 \pm 8.503 \cdot 10^{-2}$
Network centralization	$3.107 \cdot 10^{-2}$	$1.360 \cdot 10^{-1}$	$8.074 \cdot 10^{-3} \pm 1.150 \cdot 10^{-3}$
Network heterogeneity	1.842	2.520	$6.098 \cdot 10^{-1} \pm 1.242 \cdot 10^{-2}$
