# Supplementary material for: Prioritisation and Network Analysis of Crohn's Disease Susceptibility Genes
Source: PLoS One. 2014 Sep 30;9(9):e108624. doi: 10.1371/journal.pone.0108624 (PMC4182533; doi:10.1371/journal.pone.0108624)
Supplement: Table S2 — Training set. Table listing the Entrez IDs included in the training set with their literature references. (PDF) [file pone.0108624.s009.pdf]

Table S2

Entrez ID	Gene name	References
64127	NOD2	[1], [2], [3]
55054	ATG16L1	[2], [3], [4]
4843	NOS2	[2], [3], [5]
3458	IFNG	[2], [3], [6]
10125	RASGRP-1	[2], [3]
5604	MAP2K1	[7], [8]
7421	VDR	[3], [9]
1665	DBP1	[3], [10]
8767	RIPK2	[3], [11]
5605	MAP2K2	[7], [12],
56848	SPHK2	[3], [13]
468	ATF4	[14], [15], [16]
2879	GPX4	[3], [17]

References

1. Cooney R, Baker J, Brain O, Danis B, Pichulik T, et al. (2010) NOD2 stimulation induces autophagy in dendritic cells influencing bacterial handling and antigen presentation. Nat Med 16(1):90-7.

2. Marcuzzi A, Bianco AM, Girardelli M, Tommasini A, Martelossi S, et al (2013) Genetic and functional profiling of Crohn's disease: autophagy mechanism and susceptibility to infectious diseases. Biomed Res Int 2013:297501.

3. Jostins L, Ripke S, Weersma RK, Duerr RH, McGovern DP, et al. (2012) Host-microbe interactions have shaped the genetic architecture of inflammatory bowel disease. Nature 491(7422):119-24.

4. Travassos LH, Carneiro LA, Ramjeet M, Hussey S, Kim YG, et al. (2010) Nod1 and Nod2 direct autophagy by recruiting ATG16L1 to the plasma membrane at the site of bacterial entry. Nat Immunol 11(1):55-62.

5. Kraynack NC, Corey DA, Elmer HL, Kelley TJ (2002) Mechanisms of NOS2 regulation by Rho GTPase signaling in airway epithelial cells. Am J Physiol Lung Cell Mol Physiol 283(3):L604-11.

6. Sasaki T, Hiwatashi N, Yamazaki H, Noguchi M, Toyota T (1992) The role of interferon gamma in the pathogenesis of Crohn's disease. Gastroenterol Jpn 27(1):29-36.

7. Van Den Blink B, Ten Hove T, Van Den Brink GR, Peppelenbosch MP, Van Deventer SJ (2002) From Extracellular to Intracellular Targets, Inhibiting MAP Kinases in Treatment of Crohn's Disease. Ann N Y Acad Sci 973:349-58.

8. Lee JC, Lyons PA, McKinney EF, Sowerby JM, Carr EJ (2011) Gene expression profiling of CD8+ T cells predicts prognosis in patients with Crohn disease and ulcerative colitis. J Clin Invest 121(10):4170-9.

9. Simmons JD, Mullighan C, Welsh KI, Jewell DP (2000) Vitamin D receptor gene polymorphism: association with Crohn's disease susceptibility. Gut 47(2):211-4.

10. Lipinski S, Grabe N, Jacobs G, Billmann-Born S, Till A, et al. (2012) RNAi screening identifies mediators of NOD2 signaling: implications for spatial specificity of MDP recognition. Proc Natl Acad Sci U S A 109(52):21426-31.

11. Tigno-Aranjuez JT, Asara JM, Abbott DW (2010) Inhibition of RIP2's tyrosine kinase activity limits NOD2-driven cytokine responses. Genes Dev 24(23): 2666-2677.

12. Zahn A, Moehle C, Langmann T, Ehehalt R, Autschbach F, et al. (2007) Aquaporin-8 expression is reduced in ileum and induced in colon of patients with ulcerative colitis. World J Gastroenterol 13(11): 1687-1695.

13. Edmonds Y, Milstien S, Spiegel S (2011) Development of small-molecule inhibitors of sphingosine-1-phosphate signaling. Pharmacol Ther 132(3):352-60.

14. Fritz T, Niederreiter L, Adolph T, Blumberg RS, Kaser A (2011) Crohn's disease: NOD2, autophagy and ER stress converge. Gut 60:1580-1588.

15. Kaser A, Blumberg RS (2010) Endoplasmic reticulum stress and intestinal inflammation. Mucosal Immunol 3, 11-16.

16. Kaser A, Blumberg RS (2009) Endoplasmic reticulum stress in the intestinal epithelium and inflammatory bowel disease. Semin Immunol 21:156-63.

17. McGovern DP, Jones MR, Taylor KD, Marciante K, Yan X, et al. (2010) Fucosyltransferase 2 (FUT2) non-secretor status is associated with Crohn's disease. Hum Mol Genet 19 (17): 3468-3476.
