# Supplementary material for: Prioritisation and Network Analysis of Crohn's Disease Susceptibility Genes
Source: PLoS One. 2014 Sep 30;9(9):e108624. doi: 10.1371/journal.pone.0108624 (PMC4182533; doi:10.1371/journal.pone.0108624)
Supplement: Table S3 — Segregated enriched categories. Table summarising the segregated enriched categories containing STAT3, JAK2, VDR, FASLG (see section 'Results and discussion' in the main text). ATF4 and PRDM1 are not reported not being present in such categories. (PDF) [file pone.0108624.s010.pdf]

Table S3

STAT3	JAK2	VDR	FASLG
**Biological Process**<br>immune response<br>cellular defense response<br>immune system process<br>response to stimulus<br>intracellular signaling cascade<br>cell communication<br>signal transduction<br>JAK-STAT cascade<br>cellular process<br><br>apoptosis<br>**Pathway**<br>Inflammation mediated by chemokine and cytokine signaling pathway	**Biological Process**<br>cellular defense response<br>immune system process<br>response to stimulus<br>intracellular signaling cascade<br>cell communication<br>signal transduction<br>cell-cell signaling<br>JAK-STAT cascade<br>cell surface receptor<br>linked signal transduction<br>induction of apoptosis<br>cellular process<br>apoptosis<br><br>**Molecular Function**<br><br>receptor activity<br>**Pathway**<br>Inflammation mediated by chemokine and cytokine signaling pathway<br>**Protein Class**<br>receptor	**Biological Process**<br>intracellular signaling cascade<br>cell communication<br>signal transduction<br>cellular process<br>**Molecular Function**<br>receptor activity<br>**Protein Class**<br>receptor	**Biological Process**<br>immune response<br>cytokine-mediated signaling pathway<br>cellular defense response<br>immune system process<br>response to stimulus<br>intracellular signaling cascade<br>MAPKKK cascade<br>cell communication<br>signal transduction<br><br>cell-cell signaling<br>I-kappaB kinase/NF-kappaB cascade<br>response to interferon-gamma<br><br>cell surface receptor<br>linked signal transduction<br>induction of apoptosis<br>cellular process<br>apoptosis<br><br>**Molecular Function**<br>cytokine activity<br>receptor binding<br>receptor activity<br>protein binding<br>**Protein Class**<br>cytokine<br>signaling molecule
