# Supplementary material for: Prioritisation and Network Analysis of Crohn's Disease Susceptibility Genes
Source: PLoS One. 2014 Sep 30;9(9):e108624. doi: 10.1371/journal.pone.0108624 (PMC4182533; doi:10.1371/journal.pone.0108624)
Supplement: Information S1 — Supplementary Text and Supplementary Tables. (PDF) [file pone.0108624.s012.pdf]

Supporting Information

1 Large scale organisation of the network associated with disease

In this section we analyse the topological properties that characterise the large scale organisation of our network associated with disease. In section 1.1 we review and apply topological distributions and metrics to delineate the network structure; in section 1.2 we analyse the network robustness to nodes removals.

1.1 Topological distributions and metrics

One of the major goals in network theory is the identification of few general principles that govern the architecture of complex networks as they evolve in natural systems. Recently, the concept of hierarchical and modular organisation in complex networks was introduced in the context of metabolism in [1]. The authors analysed metabolic networks of 43 distinct organisms and found that they are organised into many small, highly connected topological modules that combine hierarchically into larger, less cohesive units. Important signatures of hierarchy and modularity are the scaling of the average clustering coefficient distribution and of the topological coefficient distributions [1], [2]. A formal definition of the average clustering coefficient distribution, together with definitions of the other metrics used, is reported in the Appendix. A power law scaling of the average clustering coefficient distribution indicates that proteins with a low number of connections tend to be part of highly linked regions, these communicating through few, weakly clustered hubs. This property is typical of hierarchical networks whose structure is based on successive layers of highly interconnected groups of nodes communicating through hubs. The topological coefficient distribution is defined similarly to the average clustering coefficient distribution, see Appendix, and its decrease with the number of neighbours indicates that hubs do not have more common neighbours than proteins with fewer links, this confirming a modular network organisation.

We analysed the topological properties of the disease network built from Crohn's susceptibility genes to determine if they were in agreement with the ones just described. We investigated the associated topological distributions using the Cytoscape plugin Network Analyzer [3], [4]. Typical parameters that characterised the network associated with disease, the NCBI human protein interaction network and average parameters of 30 randomised networks with the same size and number of edges as the network associated with disease are reported in Table S1; characteristic graph theoretical distributions are reported in Figure S1. The network associated with disease is composed of 28 connected components of which 1 is a giant component and presents a density value which is approximately three times higher than in the NCBI human protein interaction network, this indicating that the number of edges is high relatively to the network dimension, see Table S1. Network centralisation and heterogeneity reflect the tendency of the network to contain hub nodes, these being significantly higher than in randomised networks (see Table S1). The clustering coefficient distribution follows a power law indicating a hierarchical organisation in the disease network whereas it is not significantly fitted by a power law in the NCBI human protein interaction network, see Figures S1a, S1b. Preferential attachment together with a decrease of the topological coefficient distribution indicate a modular network organisation [1], [5], see Figures S1c, S1d. Overall both the NCBI human protein interaction network and the prioritised Crohn's disease network present topological properties that resemble the ones of other biological networks, this supporting their biological viability.

1.2 Failure-attack tolerance

Disease is often caused by perturbation in the communication between bio-molecules [6], [7]. Investigating how such changes at the local level can affect the network structure may provide insight on its robustness

and highlight which components are critical to maintain a correct functioning. A graph theoretical analysis of how nodes removal affect the overall connectedness of complex communication networks showed that topology plays an important role in generating robustness [8]. When compared to an Erdős-Rényi model, these networks were found to be robust to progressive removal of nodes, when they were randomly selected (failures), but more vulnerable to deletion of nodes playing an important role in maintaining the network's connectivity (attacks). Such resilience can be quantified by analysing how a specific network attribute is affected when disabling nodes one after the other. In particular, to investigate the robustness of the network that we associated with Crohn's disease, we monitored how the total number of interactions vary as a function of the percentage of removed nodes; the curves obtained are called co-extinctions curves. The computation was performed by applying the open source software for complex network analysis NEXCADE [7]. In Figure S2a we show a co-extinction curve representing how the number of interactions in the disease network vary when nodes are removed randomly, from the highest degree to the lowest and from the lowest to the highest degree. Random removal of nodes caused the total number of interactions to decrease approximately linearly; in contrast, removal of nodes from the highest to the lowest degree resulted in a rapid decrease in the total number of interactions leading to network fragmentation within deletion of the first 20% of nodes. The network also presents stronger resilience when the co-extinction curve, calculated when removing nodes from the lowest to the highest degree, is compared to the co-extinction curves obtained when deleting nodes in reverse or random order. We then investigated the indirect influence of node removals to secondary extinctions, these being defined as follows. By defining a node as extinct when either it is removed or it does not share any connection with another node in the network, the number of extinctions is calculated when they are either directly or indirectly caused by the current perturbation; in the former case they are called primary whereas in the latter secondary. In Figure S2b we show a co-extinction curve representing how the number of secondary extinctions vary when nodes are removed randomly, from the highest degree to the lowest and from the lowest to the highest degree. Similarly to the co-extinction curve evaluating the number of interactions, secondary extinctions present high susceptibility to hub removals; nevertheless, they appear to be more resilient when removing random nodes and nodes from lowest to highest degrees. In Figures S2c, S2d we show the same co-extinction curves when applied to an Erdős-Rényi network model with the same number of nodes and interactions as in the disease network. The co-extinction curve of the number of interactions (Figure S2c) shows an approximately linear decrease which is similar to the one observed in the corresponding curve of the disease network (Figure S2a). Conversely, removal of nodes in sorted order highlighted higher resilience of the disease network when nodes were removed from the lowest degree to the highest, whereas higher susceptibility is shown when deletion occurs in the reverse order. When comparing the secondary extinction cascade of the disease network (Figure S2b) with the one evaluated on the Erdős-Rényi model (Figure S2d), the disease network presented higher resilience to random removal of nodes, this being in contrast with the similarity of the co-extinctions curves of the number of interactions when affected by the same perturbation (Figures S2a, S2c). Similarly to the co-extinction curves associated with the number of interactions, deletion of nodes in sorted order presents higher susceptibility in the disease network than in the Erdős-Rényi model when removals occur from the highest to the lowest node degree; instead, it shows higher resilience when nodes are removed in reverse order. Overall the robustness of the network associated with disease to removal of nodes with low degree and its susceptibility to deletion of highly connected nodes reflects the key role played by hub proteins in maintaining the connectivity of this biological network.

Since in some cases SNPs have been shown to be false positives and the removal of the genes associated with their windows loci would affect the network connectivity, we tested the network robustness when removing nodes associated with the SNPs loci windows. The results are shown in Figure S3 and are compared with removal of an equivalent number of nodes per SNP when selected from the lowest to the highest degree and from the highest to the lowest degree. Robustness is evaluated as number of remaining interactions and shows higher network susceptibility to removal of nodes associated with the SNPs loci

windows than to removal of nodes selected from the lowest to the highest degree; conversely, the network is more robust to removal of nodes associated with the SNPs loci windows than when removing nodes from the highest to the lowest degree.

Appendix

In what follows we list the formal definitions of the graph theoretical metrics that we applied in our analysis.

Formal definitions of graph measures

Average clustering coefficient distribution
The clustering coefficient of a node is defined by

$$C_i = \frac{2n_i}{k_i(k_i - 1)} \tag{1}$$

where k_i is the degree of node i and n_i denotes the number of edges between neighbours of node i [4]. The average clustering coefficient distribution $C(k)$ is evaluated by calculating the average of the clustering coefficients for all nodes n with exactly k neighbours by varying k. A power law scaling of $C(k)$ indicates that proteins with a low number of connections tend to be part of highly linked regions, these communicating through few, weakly clustered hubs.

Topological coefficient distribution
The topological coefficient T_i of a node i with k_i neighbours estimates the tendency of the node to have shared neighbours with other nodes; this is defined as follows

$$T_i = \frac{< J(i,j) >}{k_i} \tag{2}$$

where $J(i,j)$ is the number of neighbours shared between the nodes i and j, plus one if there is a direct link between i and j [4], [9]. The topological coefficient distribution $T(k)$ is defined similarly to the average clustering coefficient distribution and its decrease with the number of neighbours indicates that hubs do not have more common neighbours than proteins with fewer links, this confirming a modular network organisation.

Number of hubs
Following the definition suggested in [10], we define nodes as hubs if their nodal degree exceeds the average degree of the network, namely

$$\text{N. Hubs} = \sum_{i}^{N} \Big[k_i > \langle k \rangle \Big].$$

Average (or characteristic) path length
The average path length L is defined as the average distance between any two nodes in the network, where the distance between two particular nodes is the minimum number of edges needed to connect them.

Network density

The density of a network D is given by the ratio of the number of its edges E to the number of all possible edges, these being $\binom{N}{2}$; it follows

$$D = \frac{2E}{N(N-1)}.$$

A network comprising isolated nodes only has a density of 0; conversely, the density of a clique is 1.

Network diameter
The network diameter is given by the maximum of all shortest path lengths. In case the network is composed of several connected components, its diameter is defined as the maximum of the diameters calculated for each connected component.

Network centralisation
The network centralisation is an index of the connectivity distribution which highlights if the network has a topology which *resembles* a star, in which case its centralisation is close to 1, or it is decentralised, in which case its centralisation is close to 0 [11]. Given a network of size N, degree vector $\mathbf{k}$ and density d, the network centralisation is given by

$$Centralisation = \frac{N}{N-2}\left(\frac{max(\mathbf{k})}{N-1} - d\right) \approx \frac{max(\mathbf{k})}{N} - d.$$

Network heterogeneity
The network heterogeneity is an index based on the variance of the connectivity and equals the coefficient of variation of the connectivity distribution, namely

$$Heterogeneity = \frac{\sqrt{\sigma(\mathbf{k})}}{\mu(\mathbf{k})},$$

where $\mu(\mathbf{k})$ and $\sigma(\mathbf{k})$ are respectively the mean value and the variance of the degree vector $\mathbf{k}$ [11].

Giant component
A giant component is a connected subgraph that contains the majority of the entire graph's nodes.

Erdős-Rényi model
The Erdős-Rényi model of a random network can be defined in two variants. In the first variant the number of nodes N and the probability that each possible edge between any two nodes is present is fixed to a value p. If each node is connected to an average of z edges, then $p = z/(N-1)$ and, for large N, $p \approx z/N$. Denoting by k the vertex degree, its probability distribution is given by

$$p_k = \binom{N}{k} p^k (1-p)^{N-k} \simeq \frac{z^k e^{-z}}{k!},$$

where the second equality is exact when N is large so that the node degrees follow the Poisson distribution within such limit [12]. In the second variant the number of nodes N and the number of edges E are defined and a graph is chosen uniformly at random from all possible graphs with N nodes and E edges. In this work we applied the second variant to compare a network comprising realistic interactions with randomly generated networks with the same number of nodes and edges.

References

1. Ravasz E, Somera AL, Mongru DA, Oltvai ZN, Barabasi AL (2002) Hierarchical organization of modularity in metabolic networks. Science 30;297(5586):1551-5.

2. Stelzl U, Worm U, Lalowski M, Haenig C, Brembeck FH, et al. (2005) A human protein-protein interaction network: a resource for annotating the proteome. Cell 122(6):957-68.

3. Assenov Y, Ramirez F, Schelhorn SE, Lengauer T, Albrecht M (2008) Computing topological parameters of biological networks. Bioinformatics 24, 2, 282-284.

4. Doncheva NT, Assenov Y, Domingues FS, Albrecht M (2012) Topological analysis and interactive visualization of biological networks and protein structures. Nat Protoc 7(4):670-85.

5. Sengupta U, Ukil S, Dimitrova N, Agrawal S (2009) Expression-Based Network Biology Identifies Alteration in Key Regulatory Pathways of Type 2 Diabetes and Associated Risk/Complications. PLoS ONE 4(12): e8100.

6. Nussinov R, Panchenko AR, Przytycka T (2011) Physics approaches to protein interactions and gene regulation. Phys Biol 8(3):030301.

7. Yadav G, Babu S (2012) NEXCADE: Perturbation Analysis for Complex Networks. PLoS ONE 7(8): e41827.

8. Albert R, Jeong H, Barabasi AL (2000) Error and attack tolerance of complex networks. Nature 406, 378-382.

9. Assenov Y, Ramirez F, Schelhorn SE, Lengauer T, Albrecht M (2008) Computing topological parameters of biological networks. Bioinformatics 24, 2, 282-284.

10. van Wijk BC, Stam CJ, Daffertshofer A (2010) Comparing brain networks of different size and connectivity density using graph theory. PLoS One 5(10):e13701.

11. Dong J, Horvath S (2007) Understanding Network Concepts in Modules. BMC Syst Biol 1:24.

12. Newman ME, Strogatz SH, Watts DJ (2001) Random graphs with arbitrary degree distributions and their applications. Phys Rev E 64, 026118.
